# Novel Pyridyl–Oxazole Carboxamides: Toxicity Assay Determination in Fungi and Zebrafish Embryos

**DOI:** 10.3390/molecules26133883

**Published:** 2021-06-25

**Authors:** Shu Chen, Dong-Lin Zhang, Chao-Li Ren, Wen-Qian Zou, Xiao-Yu Tian, Xiao-Hua Du, Cheng-Xia Tan

**Affiliations:** College of Chemical Engineering, Zhejiang University of Technology, Hangzhou 310014, China; omunyu@163.com (S.C.); z1076718730@163.com (D.-L.Z.); 18708589843@163.com (C.-L.R.); zwqxyes@163.com (W.-Q.Z.); t895745887@163.com (X.-Y.T.); duxiaohua@zjut.edu.cn (X.-H.D.)

**Keywords:** oxazole, amide, pyridine, fungicide activity, toxicity

## Abstract

Eight novel pyridyl–oxazole carboxamides were evaluated against fungi and displayed good fungicidal activities against *Botrytis cinereal* and *Rhizoctonia solani*. Preliminary bioassay results indicated that at 100 mg/L, compounds **6a**–**6e**, **6g** and **6h** exhibited 100% fungicidal activities against *Botrytis cinerea*, and the compound **6b** to *Rhizoctonia solani* at 100%. Then, the zebrafish embryo acute toxicity test was performed to assess the toxicity of **6b** and **6c**. A series of malformations appeared, when the zebrafish embryos were exposed to **6b** and **6c**, such as delayed yolk sac resorption, significant shortening of body length, pericardial edema, bending spine, lack of melanin, heart hemorrhage, head hemorrhage, delayed swim sac development, yolk malformation and head malformation. In addition, the acute toxicity of **6b** to zebrafish embryo is 4.878 mg/L, and **6c** is 6.257 mg/L.

## 1. Introduction

In the early 20th century, pesticide, as a helpful partner for agriculture, came into our lives. Lots of people realized the fact that every coin has two sides: the effective pesticides may be hazardous to human health and the environment. In recent decades, the mushrooming human population has come with a booming economy, followed by the unexpected impact from the accumulation of environmental pollution caused by abusing pesticides. The residues of pesticides slipped into the food chain quietly, through bioconcentration and biomagnification, invisibly and continuously, affecting all natural creatures.

Since quality of life has improved, people have been concerned with the pesticide residues and setting higher demands on pesticides, including insecticides, herbicides, and fungicides. Currently, it is hard to imagine the world without pesticides, which have already permeated so many aspects of our lives. Thus, toxicological study of pesticides, by helping people directly and visually recognize the toxicity of pesticides, is absolutely significant. In recent years, because of certain inherent features, such as the comparable genetic and physiological make-up of mammals, zebrafish are widely used in toxicity research as an alternative model. Since zebrafish can absorb small molecules in their environment through skin and gills [1], their embryos and larvae were commonly used in developmental toxicity research to assess toxicity, such as cardiovascular toxicity, neurotoxicity and ototoxicity [2,3,4].

**Figure 1:**
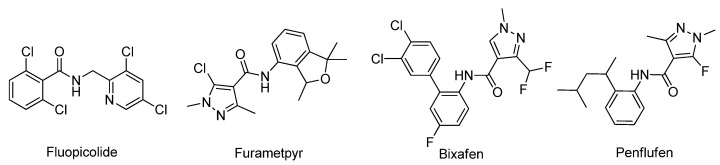
Chemical structures of Fluopicolide, Furametpyr, Bixafen and Penflufen.

**Figure 2:**
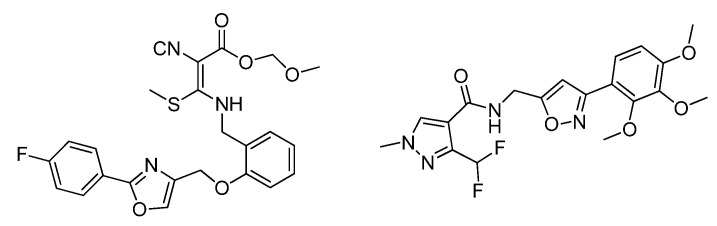
Chemical structures of oxazole derivatives.

Along with social progress and the awakening of people’s awareness of human health and environmental protection, many highly toxic pesticides were gradually eliminated by market forces, which urged researchers to search for ideal alternatives to those pesticides. Amide compounds with ideal biological activity and metabolic properties were widely used in insecticide and fungicide [5,6]. Additionally, in the process of creating green pesticides, heterocyclic pesticides have the characteristics of high activity and good selectivity, thus becoming one of the hot spots in the development of pesticide lead compounds [7,8,9,10,11]. Since the 20th century, many highly active amide-containing fungicidal drugs containing heterocyclic structures [12,13,14,15] have been developed (Figure 1). Nitrogen-containing heterocyclic compounds have various structural forms and good biological activity. Thus, the compound is widely used in the field of pesticides. At the same time, pyridine derivatives [16,17,18,19,20,21] and oxazole derivatives [22,23,24,25,26,27] (Figure 2) have also attracted attention as important nitrogen-containing heterocyclic compounds.

**Figure 3:**
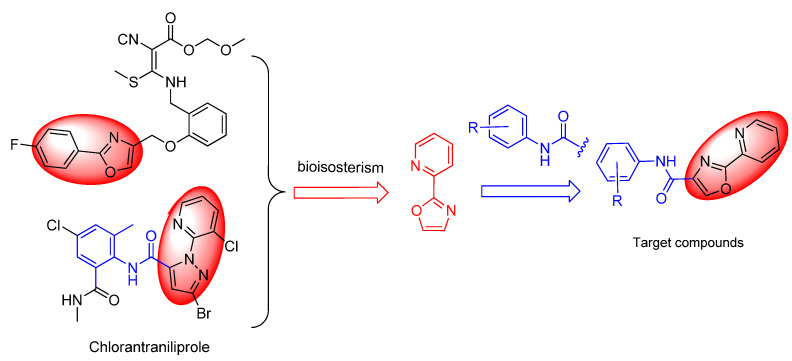
Design strategy of target compounds.

**Scheme 1:**
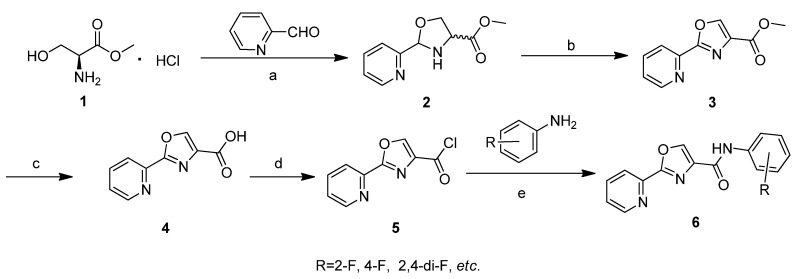
Synthetic route of target compounds. Reagents and conditions: (a) CH_2_Cl_2_, Et3N, Na_2_SO_4_, rt for 10 h [28,29]; (b) CH_2_Cl_2_, DBU, CCl_3_Br, 0 °C for 12 h [29]; (c) CH_3_OH, OH^−^, rt for 0.5 h; H^+^, rt for 1 h; (d) SOCl_2_, Reflux for 2 h; (e) CH_2_Cl_2_, Et_3_N, rt for 2 h.

Chlorantraniliprole was used as the lead compound, relating to the phenylcyclobioxazole structure of oxazole derivatives, while the principle of bioelectronic isostery was used to obtain the pyridixazole structure, introduce the benzamide structure and, finally, design and synthesize several pyridines bioxazole amide compounds (Figure 3). The synthetic route is shown in Scheme 1. Furthermore, their fungicidal activities and toxicity test on zebrafish embryos were studied.

## 2. Results and Discussion

### 2.1. Biological Activities of Target Compounds

The fungicidal activity test results of the compounds **6a**–**6h** against *Botrytis cinereal* and *Rhizoctonia solani* are shown in Table 1 and Table 2. It can be seen that the target compounds had a high inhibitory effect on *Botrytis cinereal,* of which at a concentration of 100 mg/L, **6f** had a 90% inhibitory rate, and **6a**–**6e**, **6g** and **6h** were 100%. Additionally, at a concentration of 100 mg/L, the inhibition rates of **6a**, **6e**, and **6h** against *Rhizoctonia solani* were 90%, and **6b** was 100%. According to the data analysis in Table 1 and Table 2, the results indicated that the inhibition rate of fluorine substitution was higher than that of chlorine substitution and methyl substitution when the benzene ring was substituted.

It can be seen from Table 1 and Table 2 that at a concentration of 50 mg/L, the inhibition rates of **6c** (79.12%) and **6f** (66.77%) on *Rhizoctonia solani* were better than azoxystrobin, and **6c**–**6f** (62.06–66.47%) and **6h** (64.61%) on *Botrytis cinereal* were better than azoxystrobin. According to the data in Table 2, it can be seen that at a concentration of 50 mg/L, when the benzene ring was substituted, the inhibition rate of di-fluorine substitution was higher than mono-fluorine substitution against *Rhizoctonia solani*. On *Botrytis cinereal,* the inhibition rate of its di-substitution was higher than that of mono-substitution when the substituents on the benzene ring were fluorine and methyl.

### 2.2. Toxicity to Zebrafish Embryos

According to the data mentioned above, comparing their fungicidal activities, we chose compound **6b** and **6c** to study the lethal and teratogenic effects exposure on zebrafish embryos from 6 to 96 hpf.

**Figure 4:**
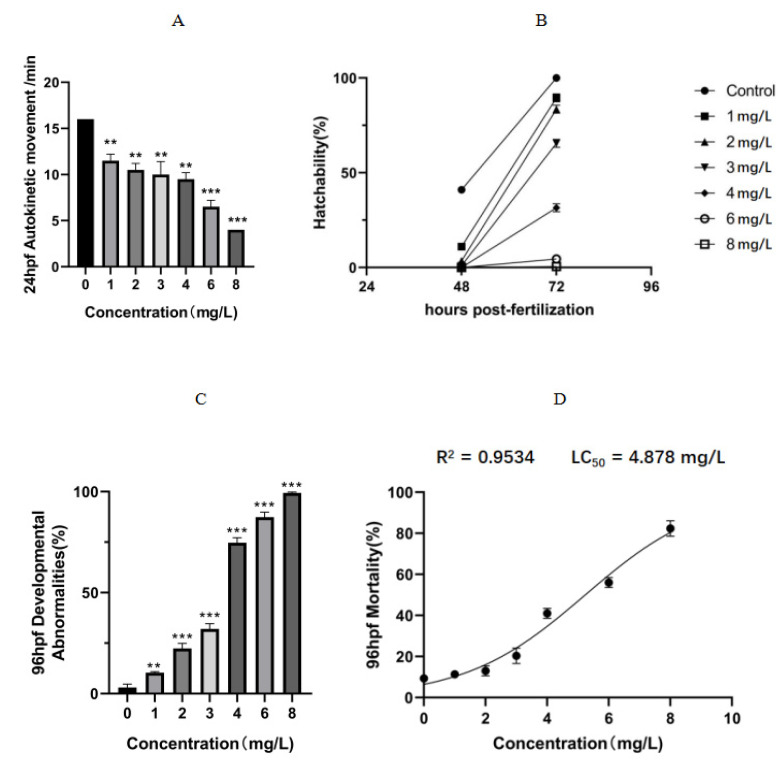

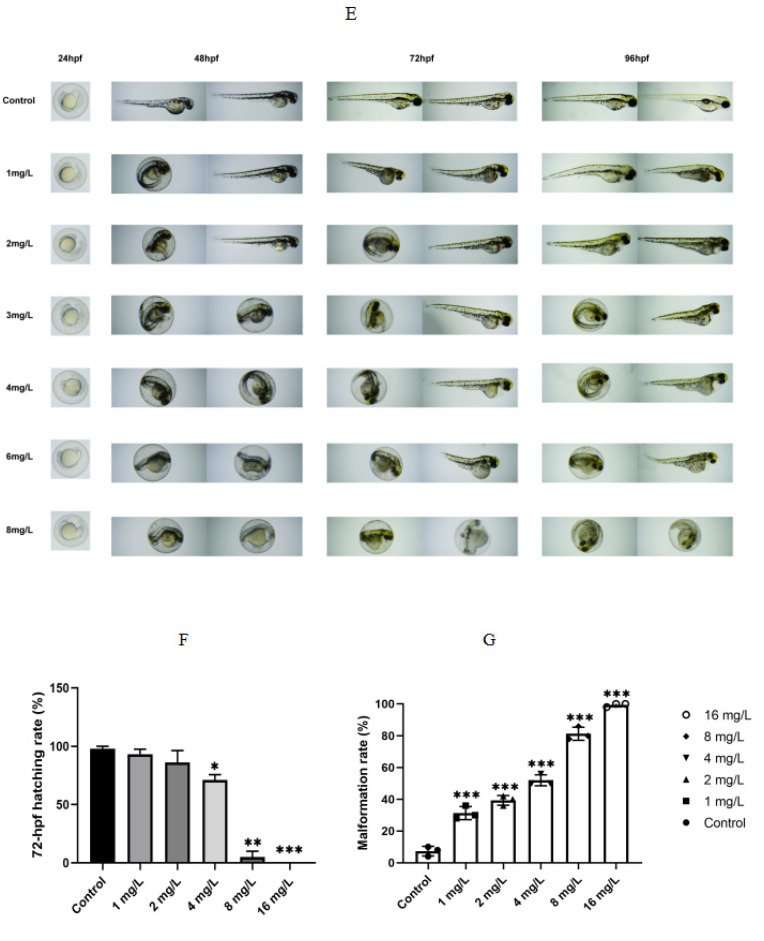

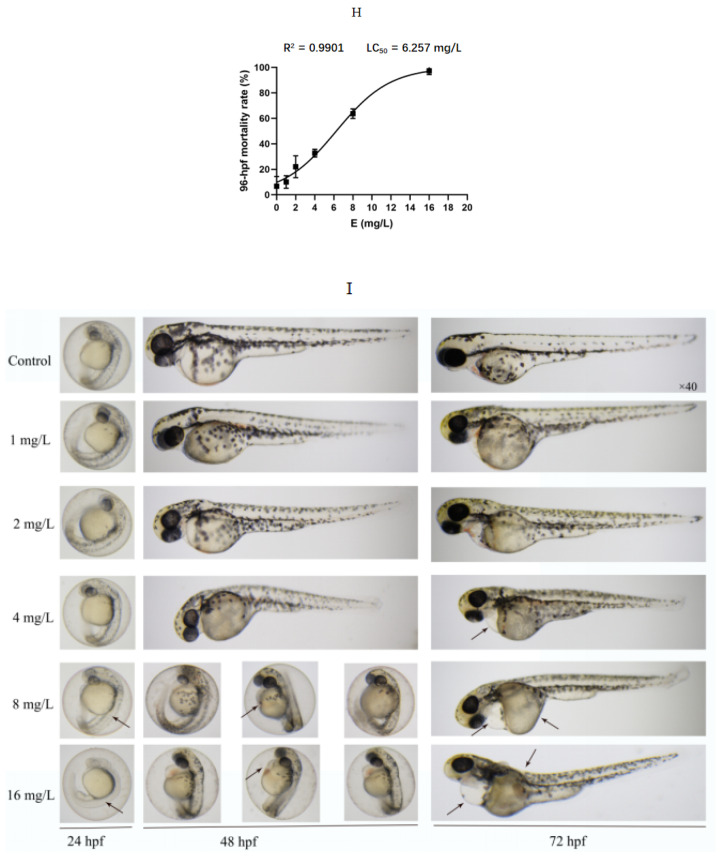
The effects of compound **6b** and **6c** exposure to zebrafish embryos. Note: (**A**) Zebrafish embryos autokinetic movement per minute after exposure to compound **6b** from 6 to 24 hpf; (**B**) The hatchability of zebrafish embryos exposed to **6b** from 24 to 96 hpf; (**C**) The developmental abnormalities of zebrafish embryos exposed to **6b** at 96 hpf; (**D**) The mortality of zebrafish embryos exposed to **6b** at 96 hpf; (**E**) The main phenotypic changes of zebrafish embryos and larvae under **6b** exposure from 24 to 96 hpf; (**F**) The hatching rate of zebrafish embryos exposed to **6c** at 72 hpf; (**G**) The malformation rate of zebrafish embryos and larvae under **6c** exposure at 96 hpf; (**H**) The mortality of zebrafish embryos exposed to **6c** at 96 hpf; (**I**) The main phenotypic changes of zebrafish embryos and larvae under **6c** exposure from 24 to 72 hpf. “*” represents significant differences at * *p* ≤ 0.05, ** *p* ≤ 0.01 and *** *p* ≤ 0.001 by one-way ANOVA followed by a Dunnet test.

At 24 hpf of **6b** (Figure 4A), the autonomous movement of zebrafish embryo within 1 min was obviously inhibited, and there was a concentration-dependent effect. Among them, the 3 and 4 mg/L groups had a decrease in the autonomous movement of the embryo (*p* < 0.01), and in the 6 and 8 mg/L treatment groups, the number of autonomous movements of embryos was even less (*p* < 0.001). At 48 hpf (Figure 4B), compared to the control group, the hatching rate of the **6b** drug-exposed group was significantly suppressed. At 72 hpf (Figure 4B), under **6b** concentration of 6 mg/L, the hatching rate was about 5%, and under 8 mg/L exposure, the hatching rate was 0%. At 96 hpf of **6b** (Figure 4C), the zebrafish malformation rate gradually increased with a concentration-dependency (*p* < 0.01 and *p* < 0.001). The main phenotypic changes of zebrafish under **6b** acute exposure (Figure 4E) were shortened body length, delayed tail development, delayed eye development, yolk cyst and even deformity, and there was a concentration-dependent effect. At the 48–96 hpf, a series of malformations appeared, such as delayed yolk sac resorption, significant shortening of body length, pericardial edema, bending spine, lack of melanin, heart hemorrhage, head hemorrhage, delayed swim sac development, yolk malformation and head malformation. The LC_50_ concentration of **6b** (Figure 4D) was 4.878 mg/L.

The main manifestations of acute violent poisoning of **6c** (Figure 4I) were yolk cyst, venous sinus hemorrhage, developmental delay, bending spine, hypopigmentation, pericardial cyst, and delayed swim sac development (represented by the black arrow). The pericardial edema was the most obvious. The hatching rate at 72 hpf was shown in Figure 4F. At 8 mg/L and 16 mg/L, the hatching rate was significantly reduced (*p* < 0.05, *p* <0.01, *p* < 0.001). The malformation rate at 96 hpf was in Figure 4G. In the high-concentration exposure group, the malformation rate was significantly increased and showed a concentration-dependent (*p* < 0.001). Due to the impact of incubation and stillbirth, the mortality rate at 96 hpf was counted (Figure 4H), and the LC_50_ concentration was 6.257 mg/L.

Comparing the lethal and teratogenic effects of **6b** and **6c,** we found that the toxicity of **6b** to zebrafish embryos was higher than that of **6c**. Therefore, we speculated that the structure of di-substitution was more beneficial than mono-substitution to reduce the toxicity to zebrafish embryos.

## 3. Materials and Methods

### 3.1. Materials

The compounds **6a**–**6h** were confirmed by ^1^H-NMR, ^13^C-NMR and HRMS shown in Table 3. ^1^H-NMR and ^13^C-NMR spectra were measured on an NMR spectrometer (Bruker 500 MHz, Fallanden, Switzerland); high-resolution electrospray mass spectra (HR–ESI–MS) were determined using an UPLC H-CLASS/QTOF G2-XS mass spectrometer (Waters, Milford, MA, USA). In order to keep the compounds away from direct sunlight, the solutions of **6b** (purity ≥ 98%) and **6c** (purity ≥ 98%), prepared in tetrahydrofuran, were stocked in −20 °C refrigeration. Additionally, diluted stock solutions were added in buffered zebrafish dechlorinated tap water (pH 6.5–7.5) to prepare nominal dosing solutions [30].

### 3.2. Fish Husbandry and Embryo Collection

The zebrafish (D. rerio) used for the experiments were from the Institute of Hydrobiology. According to standard protocols, they were raised and adapted to the laboratory with a light/dark, 14 h/10 h cycle in a circulation system with dechlorinated tap water (pH 6.5–7.5) at a constant temperature (27 ± 0.5 °C). In order to raise the conductivity to 450–1000 μS/cm, the ocean salt was added to the water [30,31].

The eggs of the zebrafish that were acquired from spawning adults in tanks overnight, with a sex ratio of 2:2, were used for exposure experiments and collected within half an hour of light exposure [30]. By means of a stereomicroscope, the fertilized and normal embryos were inspected and staged for subsequent experiments. When at 6 hpf, they were distributed into 12-well plates [32] (10 embryos per well) for exposure, and three replicate experiments were performed.

### 3.3. Ethics Statement

The Institutional Animal Care and Use Committee (IACUC) at Wenzhou Medical University approved our study plan for the proper use of zebrafish. All studies were carried out in strict accordance with the guidelines of the IACUC. All dissections were performed on ice, and all efforts were made to minimize suffering.

### 3.4. Fungicidal Activity and Toxicity Determination

The fungicidal activity was investigated on the basis of reference [33], and the results were shown in Table 1 and Table 2. Furthermore, compound **6b** and **6c** were selected to be assessed through the zebrafish embryo acute toxicity test. Based on the mortality rates of zebrafish, a series of gradient concentrations of the compound **6b** and **6c** were set according to the environmentally relevant concentrations and preliminary experiments.

Zebrafish embryos (*n* = 30) at 6 hpf were selected under a stereomicroscope, and they were exposed to the compound **6b** or **6c** from 6 to 96 hpf: control (0 mg/L of **6b**), 1, 2, 3, 4, 6, 8 mg/L of **6b**; control (0 mg/L of **6c**), 1, 2, 4, 8, 16 mg/L of **6c**. The LC_50_ (median lethal concentration) values were computed by the Boltzmann equation [34,35]. The observational indexes included hatching rate, mortality rate and malformation rate.

## 4. Conclusions

In conclusion, the bioassay results indicated that at a concentration of 100 mg/L, the target compound had a high inhibitory effect on *Botrytis cinereal,* of which **6a**–**6e**, **6g** and **6h** had a 100% inhibitory rate. Furthermore, most of the target compounds had a high inhibitory effect on *Rhizoctonia solani*. **6b** (100%) had a high inhibition rate on *Rhizoctonia solani*. Additionally, the acute toxicity of compound **6b** (4.878 mg/L) and **6c** (6.257 mg/L) indicated that **6b** and **6c** exposure had an apparent influence on the normal development of the zebrafish process. By comparing their data, we also found that the toxicity of **6b** to zebrafish embryos was higher than that of **6c**. Therefore, we could draw the conclusion that, compared with mono-substitution, the structure of di-substitution was more beneficial to reduce the toxicity to zebrafish embryos, which merits further study.

## Figures and Tables

**Table 1:** Fungicidal activities (inhibition rate/%) on *Botrytis cinereal* of compounds **6**.

Compd.	R	*Botrytis cinereal*			
		100 mg/L	50 mg/L	25 mg/L	12.5 mg/L
**6a**	2-F	100	45.88	12.36	nt
**6b**	4-F	100	52.06	41.18	30.08
**6c**	2,4-di-F	100	63.83	40.30	29.71
**6d**	2,6-di-F	100	62.06	49.41	32.94
**6e**	3-Cl	100	66.47	14.71	nt
**6f**	3-Cl-2-CH_3_	90	65.00	44.71	17.36
**6g**	4-CH_3_	100	41.76	7.65	3.83
**6h**	2,4-di-CH_3_	100	64.41	28.83	7.06
Azoxystrobin		100	40	20	0

Note: nt = not tested. All the data were determined three times.

**Table 2:** Fungicidal activities (inhibition rate/%) on *Rhizoctonia solani* of compounds **6**.

Compd.	R	*Rhizoctonia solani*			
		100 mg/L	50 mg/L	25 mg/L	12.5 mg/L
**6a**	2-F	90	47.06	29.31	17.69
**6b**	4-F	100	51.77	41.71	31.77
**6c**	2,4-di-F	80	79.12	18.24	16.77
**6d**	2,6-di-F	80	50.00	42.06	20.06
**6e**	3-Cl	90	57.90	18.83	11.77
**6f**	3-Cl-2-CH_3_	80	66.77	36.77	6.18
**6g**	4-CH_3_	70	nt	nt	nt
**6h**	2,4-di-CH_3_	90	55.30	34.41	4.41
Azoxystrobin		100	60	20	0

Note: nt = not tested. All the data were determined three times.

**Table 3:** ^1^H-NMR, ^13^C-NMR and HRMS of compounds **6a**–**6h**.

Compd.	R	^1^H-NMR	^13^C-NMR	HRMS
**6a**	2-F	^1^H-NMR (600 MHz, DMSO-*d*_6_) *δ*:9.92 (s, 1H, NH), 8.98 (s, 1H, oxazole-H), 8.77 (d, *J* = 4.8 Hz, 1H, Py-H), 8.22 (d, *J* = 7.8 Hz, 1H, Py-H), 8.07 (m, 1H, Py-H), 7.81–7.82 (m, 1H, Ph-H), 7.62–7.63 (m, 1H, Py-H), 7.34–7.35 (m, 1H, Ph-H), 7.27–7.28 (m, 2H, Ph-H)	^13^C-NMR (151 MHz, DMSO-*d*_6_) *δ*: 160.4, 158.9, 156.3, 154.6, 150.6, 145.1, 144.5, 138.2, 137.3, 127.2 (d, *J* = 7.6 Hz), 126.3 (d, *J* = 26.0 Hz), 125.5 (d, *J* = 11.9 Hz), 125.0 (d, *J* = 3.3 Hz), 123.1, 116.2 (d, *J* = 19.6 Hz)	calcd for C_15_H_11_FN_3_O_2_ ([M + H]^+^) 284.0830, found 284.0822
**6b**	4-F	^1^H-NMR (600 MHz, DMSO-*d*_6_) *δ*: 10.32 (s, 1H, NH), 8.95 (s, 1H, oxazole-H), 8.77 (d, *J* = 4.8 Hz, 1H, Py-H), 8.23 (d, *J* = 8.0 Hz, 1H, Py-H), 8.07 (td, *J* = 8.0, 1.5 Hz, 1H, Py-H), 7.84–7.87 (m, 2H, Ph-H), 7.61–7.62 (m, 1H, Py-H), 7.21 (t, *J* = 9.0 Hz, 2H, Ph-H)	^13^C-NMR (151 MHz, DMSO-*d*_6_) *δ*: 160.28, 159.79, 158.97, 158.19, 150.58, 145.25, 144.44, 138.18, 137.87, 135.10, 126.28, 123.01, 123.0, 122.9, 115.70 (d, *J* = 22.1 Hz)	calcd for C_15_H_11_FN_3_O_2_ ([M + H]^+^) 284.0830 found 284.0820
**6c**	2,4-di-F	^1^H-NMR (500 MHz, DMSO-*d*_6_) *δ*: 10.06 (s, 1H, NH), 8.97 (s, 1H, oxazole-H), 8.77 (d, *J* = 5.0, 1H, Py-H), 8.21 (d, *J* = 8.0 Hz, 1H, Py-H), 8.06–8.07 (m, 1H, Py-H), 7.71–7.72 (m, 1H, Ph-H), 7.61 (q, *J* = 3.0 Hz, 1H, Py-H), 7.39–7.40 (m, 1H, Ph-H), 7.14 (t, *J* = 4.5 Hz, 1H, Ph-H)	^13^C-NMR (126 MHz, DMSO-*d*_6_) *δ*: 159.87, 158.57, 150.09, 144.68, 144.02, 137.70, 136.73, 127.60 (d, *J* = 11.0 Hz), 125.81, 122.55, 111.33 (d, *J* = 3.4 Hz), 111.15 (d, *J* = 3.4 Hz), 104.54, 104.33 (d, *J* = 2.8 Hz), 104.13	calcd for C_15_H_10_F_2_N_3_O_2_ ([M + H]^+^) 302.0736 found 302.0731
**6d**	2,6-di-F	^1^H-NMR (600 MHz, DMSO-*d*_6_) *δ*: 10.03 (s, 1H, NH), 8.97 (s, 1H, oxazole-H), 8.77–8.79 (m, 1H, Py-H), 8.21 (d, *J* = 7.8 Hz, 1H, Py-H), 8.06–8.07 (m, 1H, Py-H), 7.72–7.73 (m, 1H, Ph-H), 7.61–7.63 (m, 1H, Py-H), 7.39–7.41 (m, 1H, Ph-H), 7.15–7.16 (m, 1H, Ph-H)	^13^C-NMR (151 MHz, DMSO-*d*_6_) δ: 160.4, 159.1, 150.6, 145.2, 144.5, 138.2, 137.2, 128.2 (d, *J* = 9.5 Hz), 126.3, 123.1, 111.7 (dd, *J* = 22.1, 3.5 Hz), 104.8 (dd, *J* = 26.6, 24.2 Hz)	calcd for C_15_H_10_F_2_N_3_O_2_ ([M + H]^+^) 302.0736, found 302.0726
**6e**	3-Cl	^1^H-NMR (600 MHz, DMSO-*d*_6_) *δ*: 10.44 (s, 1H, NH), 8.98 (s, 1H, oxazole-H), 8.77 (d, *J* = 4.8 Hz, 1H, Py-H), 8.24 (d, *J* = 7.8 Hz, 1H, Py-H), 8.07–8.08 (m, 1H, Py-H), 8.03–8.04 (m, 1H, Ph-H), 7.80 (d, *J* = 8.2, 2.4 Hz, 1H, Ph-H), 7.62–7.63 (m, 1H, Py-H), 7.40 (t, *J* = 8.2 Hz, 1H, Ph-H), 7.19–7.20 (m, 1H, Ph-H)	^13^C-NMR (126 MHz, DMSO) *δ*: 159.8, 158.8, 150.1, 144.7, 144.2, 139.8, 137.7, 137.2, 132.9, 130.3, 125.8, 123.0, 122.6, 120.0, 118.9	calcd for C_15_H_11_ClN_3_O_2_ ([M + H]^+^) 300.0534, found 300.0522
**6f**	3-Cl-2-CH3	^1^H-NMR (500 MHz, DMSO-*d*_6_) *δ*: 10.15 (s, 1H, NH), 8.95 (s, 1H, oxazole-H), 8.77 (d, *J* = 5.0 Hz, 1H, Py-H), 8.22 (d, *J* = 7.8 Hz, 1H, Py-H), 8.07–8.08 (m, 1H, Py-H), 7.62–7.63 (m, 1H, Py-H), 7.43 (dd, *J* = 7.8, 1.2 Hz, 1H, Ph-H), 7.38–7.39 (m, 1H, Ph-H), 7.26 (t, *J* = 7.8 Hz, 1H, Ph-H), 2.28 (s, 3H, CH_3_)	^13^C-NMR (126 MHz, DMSO) *δ*: 160.3, 159.1, 150.6, 145.2, 144.4, 138.2, 137.6, 137.6, 134.2, 132.0, 127.4, 127.2, 126.3, 125.7, 123.0, 15.7	calcd for C_16_H_13_ClN_3_O_2_ ([M + H]^+^) 314.0691, found 314.0690
**6g**	4-CH3	^1^H-NMR (600 MHz, DMSO-*d*_6_) *δ*: 10.12 (s, 1H, NH), 8.92 (s, 1H, oxazole-H), 8.76 (d, *J* = 4.2 Hz, 1H, Py-H), 8.24 (d, *J* = 7.8 Hz, 1H, Py-H), 8.06–8.07 (m, 1H, Py-H), 7.70 (d, *J* = 8.4 Hz, 2H, Ph-H), 7.60–7.61 (m, 1H, Py-H), 7.17 (d, *J* = 17.2 Hz, 2H, Ph-H), 2.29 (s, 3H, CH_3_)	^13^C-NMR (151 MHz, DMSO-*d*_6_) *δ*: 160.2, 158.8, 150.6, 145.3, 144.2, 138.2, 138.0, 136.2, 133.5, 129.5, 126.3, 123.1, 121.0, 21.0	calcd for C_16_H_14_N_3_O_2_ ([M + H]^+^) 280.1081, found 280.1065
**6h**	2. 4-di-CH3	^1^H-NMR (500 MHz, DMSO-*d*_6_) *δ*: 9.73 (s, 1H, NH), 8.91 (s, 1H, oxazole-H), 8.76–8.78 (m, 1H, Py-H), 8.22 (d, *J* = 8.0 Hz, 1H, Py-H), 8.06–8.08 (m, 1H, Py-H), 7.61–7.63 (m, 1H, Py-H), 7.36–7.37 (m, 1H, Ph-H), 7.10 (d, *J* = 2.0 Hz, 1H, Ph-H), 7.03 (d, *J* = 8.0 Hz, 1H, Ph-H), 2.29 (s, 3H, CH_3_), 2.23 (s, 3H, CH_3_)	^13^C-NMR (126 MHz, CDCl_3_) *δ*: 159.8, 158.3, 150.1, 144.8, 143.4, 137.6, 137.4, 134.9, 132.9, 132.2, 130.9, 126.6, 125.7, 125.3, 20.5, 17.6	calcd for C_17_H_16_N_3_O_2_ ([M + H]^+^) 294.1237, found 294.1226

## Data Availability

The data presented in this study are available on request from the corresponding author.

## Sample Availability

Samples of the compounds **6b** and **6c** are available from the authors.
